# Retraction: Mapping C-Terminal Transactivation Domains of the Nuclear HER Family Receptor Tyrosine Kinase HER3

**DOI:** 10.1371/journal.pone.0267745

**Published:** 2022-04-25

**Authors:** The PLOS ONE Editors

After this article [1] was published, the University of Wisconsin-Madison investigated this work and concluded that there were western blot duplications in Figs 1B and 1C, and that the first author falsified or fabricated western blot data reported in Figs 6B, 6C, and 7A. A following analysis conducted by the U.S. Department of Health and Human Services Office of Research Integrity (ORI) corroborated the findings of falsified or fabricated data in Figs 6B, 6C and 7A (see [2] for details).

The *PLOS ONE* Editors retract this article due to the concerns identified in these investigations pertaining to the integrity of research data reporting.

TMB, MI, MJW, MMS, and DLW agreed with retraction and apologize for the issues with the published article. TMB stands by the article’s findings. NL either could not be reached or did not respond directly.
